# Supplementary material for: Impact of hepatitis E virus host- and virus-derived insertions on the antiviral response in primary human hepatocytes
Source: bioRxiv. 2026 Sep 17:2026.09.15.750637. Preprint. [Version 1] doi: 10.64898/2026.09.15.750637 (PMC13596330; doi:10.64898/2026.09.15.750637)
Supplement: Supplementary file 1 [file media-1.pdf]

Supplementary information to:

Impact of hepatitis E virus host- and virus-derived insertions on the antiviral response in primary human hepatocytes

Sarah Schlienkamp[1,2], Maximilian K. Nocke[2,3,4], Rainer G. Ulrich[5], Volker Kinast[6], Eike Steinmann[1,2,7,#], Daniel Todt[2,3,4,#]

[1]Department of Molecular and Medical Virology, Ruhr University Bochum, Bochum, Germany

[2]Hepatitis E Virus Research Hub (HepE-Hub), Bochum, Germany

[3]Department of Translational and Computational Infection Research (TRACiR), Ruhr University Bochum, Bochum, Germany

[4]European Virus Bioinformatics Center (EVBC), Jena, Germany

[5]Institute of Novel and Emerging Infectious Diseases, Friedrich-Loeffler-Institut, Greifswald-Insel Riems, Germany

[6]Institute for Medical Microbiology and Virology, Carl von Ossietzky University Oldenburg, Oldenburg, Germany

[7]German Centre for Infection Research (DZIF), External Partner Site, Bochum, Germany

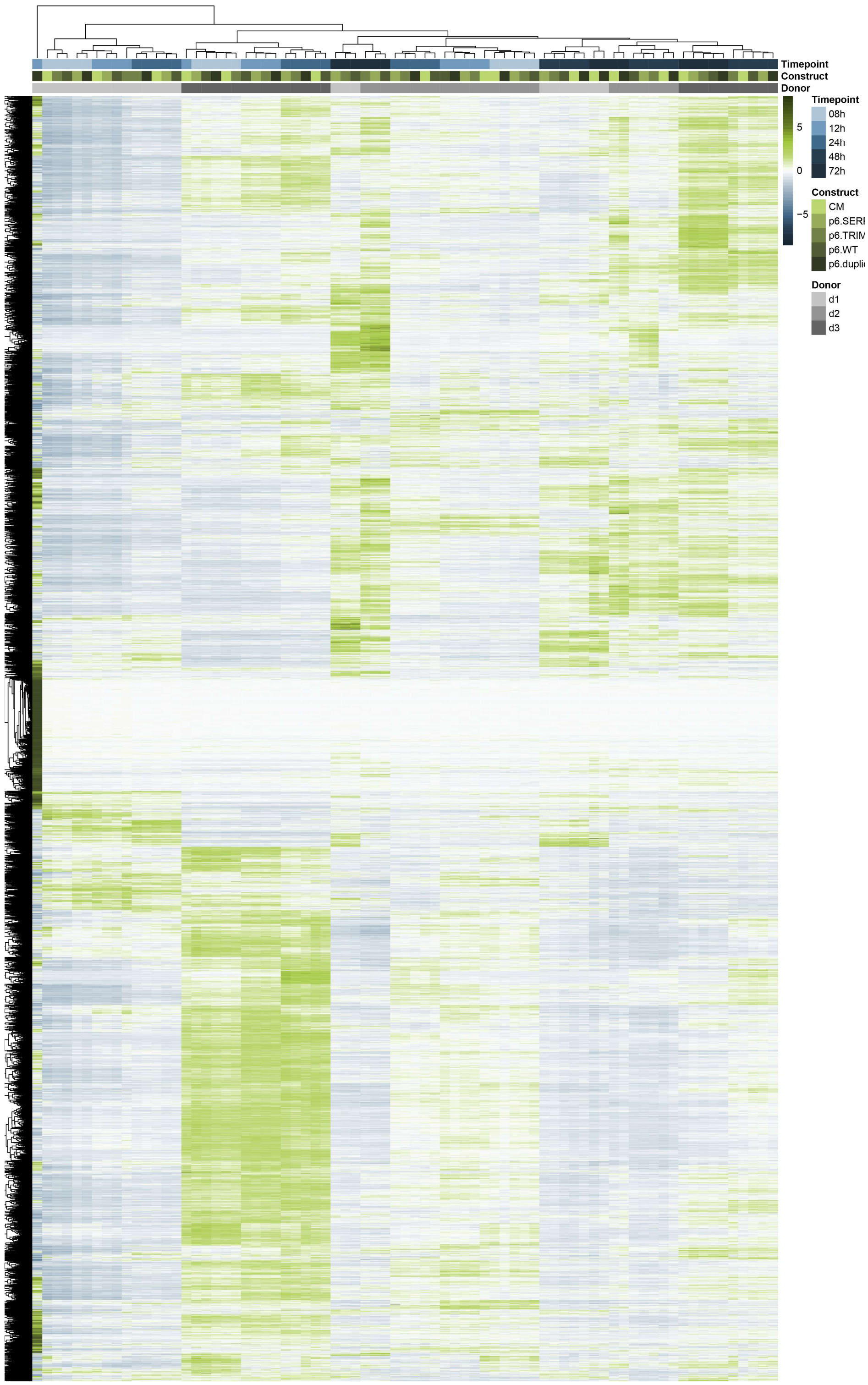

Figure S1: Clustering of normalized gene expression values of complete dataset. Presented values are z-scores of transcripts per million (TPM) clustered by rows and columns.

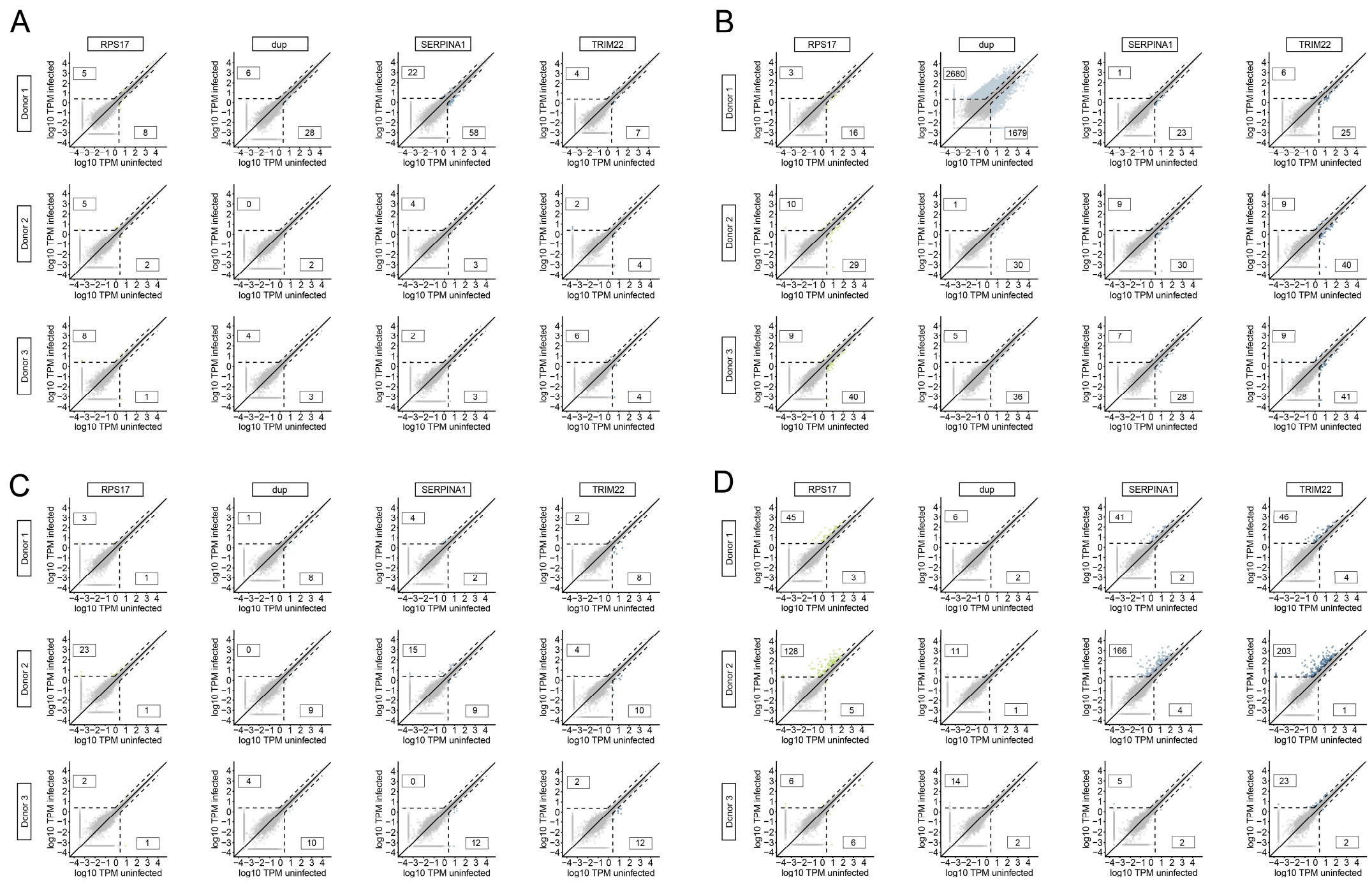

Figure S2: Virus-induced differential gene expression from 8 to 48 hours post infection. Differentially expressed genes in infected (y-axis) PHH samples compared to the respective mock-infected control (x-axis) at 8 h p.i. **(A)**, 12 h p.i. **(B)**, 24 h p.i. **(C)** and 48 h p.i. **(D)**. Rows represent differential expression upon infection with HVR insertion variants per individual donor. Dashed lines depict cutoffs of at least 3-fold change in expression and 2.47 TPM before or after infection. Included counts of DEGs above the cutoffs (colored dots) represent robustly up- or downregulated genes. Abbreviations: PHH, primary human hepatocytes; h p.i., hours post infection; HVR, hypervariable region; TPM, transcripts per million; DEGs, differentially expressed genes.

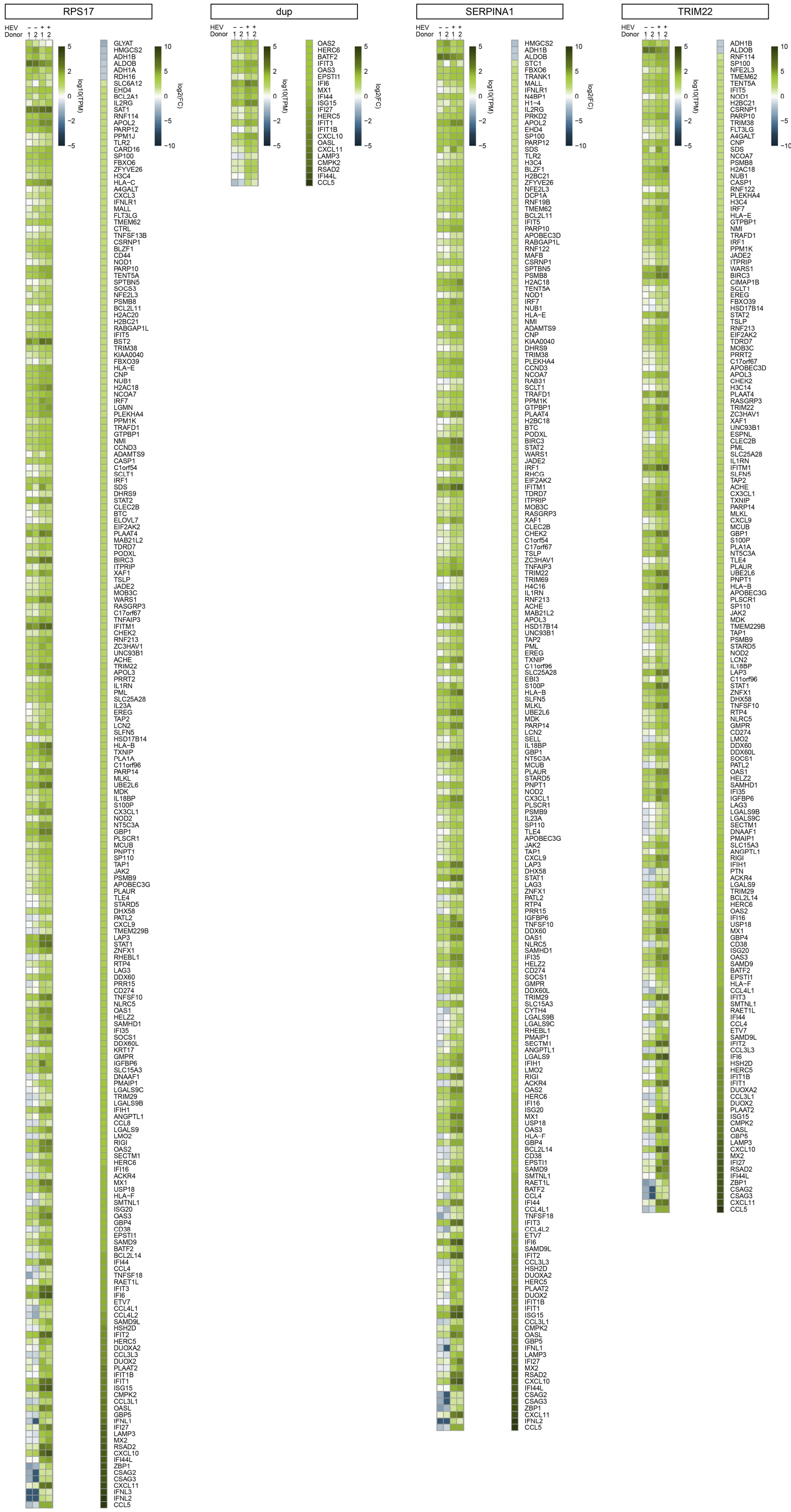
RPS17
HEV – – + +
Donor 1 2 1 2
5
2
0
−2
−5
log10(TPM)
10
5
0
−5
−10
log2(FC)
GLYAT
HMGCS2
ADH1B
ALDOB
ADH1A
RDH16
SLC6A12
EHD4
BCL2A1
IL2RG
SAT1
RNF114
APOL2
PARP12
PPM1J
TLR2
CARD16
SP100
FBXO6
ZFYVE26
H3C4
HLA−C
A4GALT
CXCL3
IFNLR1
MALL
FLT3LG
TMEM62
CTRL
TNFSF13B
CSRNP1
BLZF1
CD44
NOD1
PARP10
TENT5A
SPTBN5
SOCS3
NFE2L3
PSMB8
BCL2L11
H2AC20
H2BC21
RABGAP1L
IFIT5
BST2
TRIM38
KIAA0040
FBXO39
HLA−E
CNP
NUB1
H2AC18
NCOA7
IRF7
LGMN
PLEKHA4
PPM1K
TRAFD1
GTPBP1
NMI
CCND3
ADAMTS9
CASP1
C1orf54
SCLT1
IRF1
SDS
DHRS9
STAT2
CLEC2B
BTC
ELOVL7
EIF2AK2
PLAAT4
MAB21L2
TDRD7
PODXL
BIRC3
ITPRIP
XAF1
TSLP
JADE2
MOB3C
WARS1
RASGRP3
C17orf67
TNFAIP3
IFITM1
CHEK2
RNF213
ZC3HAV1
UNC93B1
ACHE
TRIM22
APOL3
PRRT2
IL1RN
PML
SLC25A28
IL23A
EREG
TAP2
LCN2
SLFN5
HSD17B14
HLA−B
TXNIP
PLA1A
C11orf96
PARP14
MLKL
UBE2L6
MDK
IL18BP
S100P
CX3CL1
NOD2
NT5C3A
GBP1
PLSCR1
MCUB
PNPT1
SP110
TAP1
JAK2
PSMB9
APOBEC3G
PLAUR
TLE4
STARD5
DHX58
PATL2
CXCL9
TMEM229B
LAP3
STAT1
ZNFX1
RHEBL1
RTP4
LAG3
DDX60
PRR15
CD274
TNFSF10
NLRC5
OAS1
HELZ2
SAMHD1
IFI35
SOCS1
DDX60L
KRT17
GMPR
IGFBP6
SLC15A3
DNAAF1
PMAIP1
LGALS9C
TRIM29
LGALS9B
IFIH1
ANGPTL1
CCL8
LGALS9
LMO2
RIGI
OAS2
SECTM1
HERC6
IFI16
ACKR4
MX1
USP18
HLA−F
SMTNL1
ISG20
OAS3
GBP4
CD38
EPSTI1
SAMD9
BATF2
BCL2L14
IFI44
CCL4
TNFSF18
RAET1L
IFIT3
IFI6
ETV7
CCL4L1
CCL4L2
SAMD9L
HSH2D
IFIT2
HERC5
DUOXA2
CCL3L3
DUOX2
PLAAT2
IFIT1B
IFIT1
ISG15
CMPK2
CCL3L1
OASL
GBP5
IFNL1
IFI27
LAMP3
MX2
RSAD2
CXCL10
IFI44L
ZBP1
CSAG2
CSAG3
CXCL11
IFNL3
IFNL2
CCL5
dup
HEV – – + +
Donor 1 2 1 2
5
2
0
−2
−5
log10(TPM)
10
5
0
−5
−10
log2(FC)
OAS2
HERC6
BATF2
IFIT3
OAS3
EPSTI1
IFI6
MX1
IFI44
ISG15
IFI27
HERC5
IFIT1
IFIT1B
CXCL10
OASL
CXCL11
LAMP3
CMPK2
RSAD2
IFI44L
CCL5
SERPINA1
HEV – – + +
Donor 1 2 1 2
5
2
0
−2
−5
log10(TPM)
10
5
0
−5
−10
log2(FC)
HMGCS2
ADH1B
ALDOB
STC1
FBXO6
TRANK1
MALL
IFNLR1
N4BP1
H1−4
IL2RG
PRKD2
APOL2
EHD4
SP100
PARP12
SDS
TLR2
H3C4
BLZF1
H2BC21
ZFYVE26
NFE2L3
DCP1A
RNF19B
TMEM62
BCL2L11
IFIT5
PARP10
APOBEC3D
RABGAP1L
RNF122
MAFB
CSRNP1
SPTBN5
PSMB8
H2AC18
TENT5A
NOD1
IRF7
NUB1
HLA−E
NMI
ADAMTS9
CNP
KIAA0040
DHRS9
TRIM38
PLEKHA4
CCND3
NCOA7
RAB31
SCLT1
TRAFD1
PPM1K
GTPBP1
PLAAT4
H2BC18
BTC
PODXL
BIRC3
STAT2
WARS1
JADE2
IRF1
RHCG
EIF2AK2
IFITM1
TDRD7
ITPRIP
MOB3C
RASGRP3
XAF1
CLEC2B
CHEK2
C1orf54
C17orf67
TSLP
ZC3HAV1
TNFAIP3
TRIM22
TRIM69
H4C16
IL1RN
RNF213
ACHE
MAB21L2
APOL3
HSD17B14
UNC93B1
TAP2
PML
EREG
TXNIP
C11orf96
SLC25A28
EBI3
S100P
HLA−B
SLFN5
MLKL
UBE2L6
MDK
PARP14
LCN2
SELL
IL18BP
GBP1
NT5C3A
MCUB
PLAUR
STARD5
PNPT1
NOD2
CX3CL1
PLSCR1
PSMB9
IL23A
SP110
TLE4
APOBEC3G
JAK2
TAP1
CXCL9
LAP3
DHX58
STAT1
LAG3
ZNFX1
PATL2
RTP4
PRR15
IGFBP6
TNFSF10
DDX60
OAS1
NLRC5
SAMHD1
IFI35
HELZ2
CD274
SOCS1
GMPR
DDX60L
TRIM29
SLC15A3
CYTH4
LGALS9B
LGALS9C
RHEBL1
PMAIP1
SECTM1
ANGPTL1
LGALS9
IFIH1
LMO2
RIGI
ACKR4
OAS2
HERC6
IFI16
ISG20
MX1
USP18
OAS3
HLA−F
GBP4
BCL2L14
CD38
EPSTI1
SAMD9
SMTNL1
RAET1L
BATF2
CCL4
IFI44
CCL4L1
TNFSF18
IFIT3
CCL4L2
ETV7
IFI6
SAMD9L
IFIT2
CCL3L3
HSH2D
DUOXA2
HERC5
PLAAT2
DUOX2
IFIT1B
IFIT1
ISG15
CCL3L1
CMPK2
OASL
GBP5
IFNL1
LAMP3
IFI27
MX2
RSAD2
CXCL10
IFI44L
CSAG2
CSAG3
ZBP1
CXCL11
IFNL2
CCL5
TRIM22
HEV – – + +
Donor 1 2 1 2
5
2
0
−2
−5
log10(TPM)
10
5
0
−5
−10
log2(FC)
ADH1B
ALDOB
RNF114
SP100
NFE2L3
TMEM62
TENT5A
IFIT5
NOD1
H2BC21
CSRNP1
PARP10
TRIM38
FLT3LG
A4GALT
CNP
SDS
NCOA7
PSMB8
H2AC18
NUB1
CASP1
RNF122
PLEKHA4
H3C4
IRF7
HLA−E
GTPBP1
NMI
TRAFD1
IRF1
PPM1K
JADE2
ITPRIP
WARS1
BIRC3
CIMAP1B
SCLT1
EREG
FBXO39
HSD17B14
STAT2
TSLP
RNF213
EIF2AK2
TDRD7
MOB3C
PRRT2
C17orf67
APOBEC3D
APOL3
CHEK2
H3C14
PLAAT4
RASGRP3
TRIM22
ZC3HAV1
XAF1
UNC93B1
ESPNL
CLEC2B
PML
SLC25A28
IL1RN
IFITM1
SLFN5
TAP2
ACHE
CX3CL1
TXNIP
PARP14
MLKL
CXCL9
MCUB
GBP1
S100P
PLA1A
NT5C3A
TLE4
PLAUR
UBE2L6
PNPT1
HLA−B
APOBEC3G
PLSCR1
SP110
JAK2
MDK
TMEM229B
TAP1
PSMB9
STARD5
NOD2
LCN2
IL18BP
LAP3
C11orf96
STAT1
ZNFX1
DHX58
TNFSF10
RTP4
NLRC5
GMPR
CD274
LMO2
DDX60
DDX60L
SOCS1
PATL2
OAS1
HELZ2
SAMHD1
IFI35
IGFBP6
LAG3
LGALS9B
LGALS9C
SECTM1
DNAAF1
PMAIP1
SLC15A3
ANGPTL1
RIGI
IFIH1
PTN
ACKR4
LGALS9
TRIM29
BCL2L14
HERC6
OAS2
IFI16
USP18
MX1
GBP4
CD38
ISG20
OAS3
SAMD9
BATF2
EPSTI1
HLA−F
CCL4L1
IFIT3
SMTNL1
RAET1L
IFI44
CCL4
ETV7
SAMD9L
IFIT2
CCL3L3
IFI6
HSH2D
HERC5
IFIT1B
IFIT1
DUOXA2
CCL3L1
DUOX2
PLAAT2
ISG15
CMPK2
OASL
GBP5
LAMP3
CXCL10
MX2
IFI27
RSAD2
IFI44L
ZBP1
CSAG2
CSAG3
CXCL11
CCL5

Figure S3: Gene regulation of differentially expressed gene intersections between donor 1 and 2 for each HVR insertion variant. Log_{10} TPMs for infected and uninfected samples at 72 h p.i. are presented next to their corresponding log_2 FCs. Heatmap values are ordered by decreasing log_2 FC. Abbreviations: TPM, transcripts per million; FC, fold change.

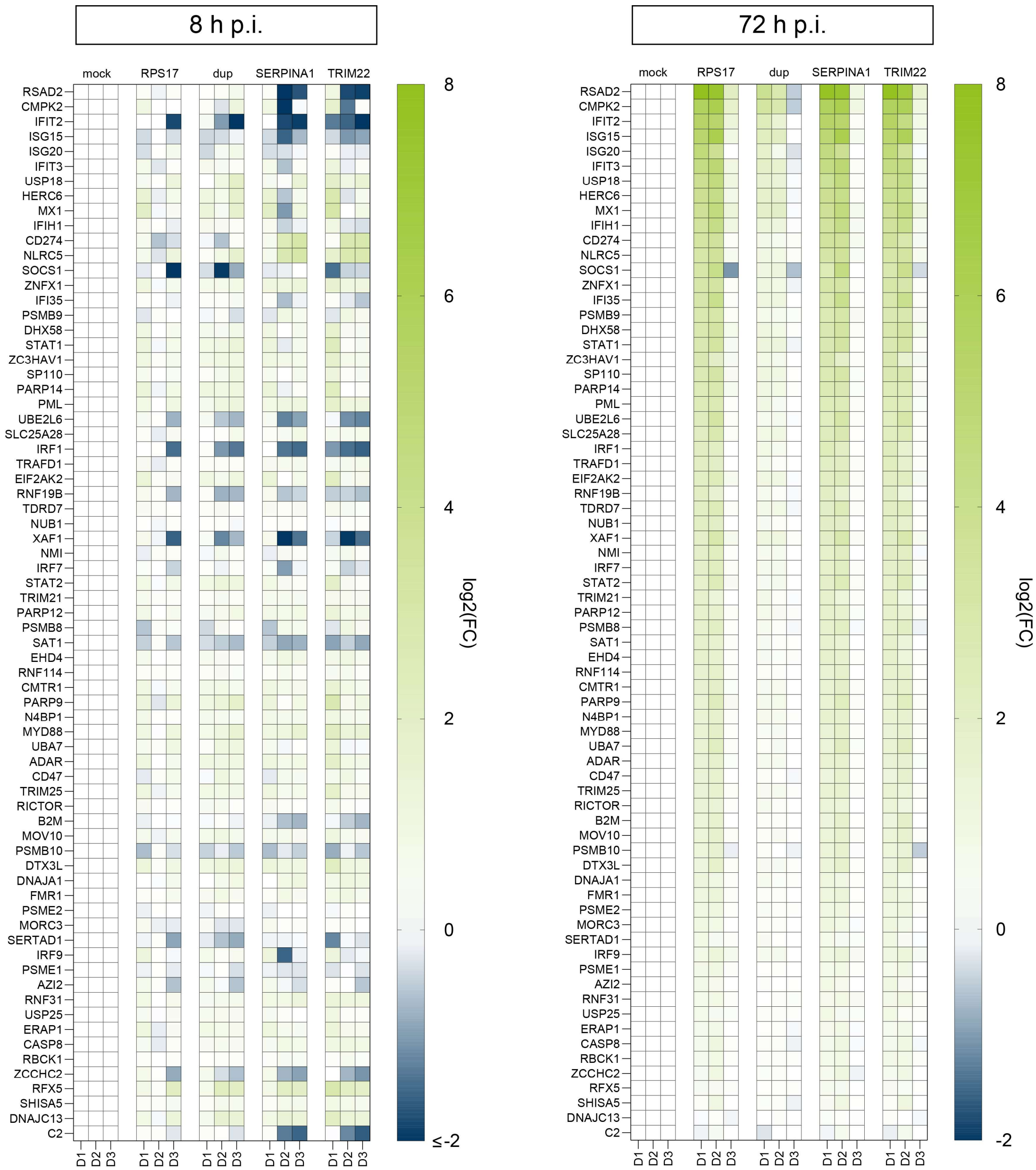

Figure S4: Core-ISG induction upon infection with HVR insertion variants. Heatmap with detailed deregulation of individual core-ISGs [1] in PHHs from three independent donors (labeled below) at 8 and 72 h p.i. with different HVR insertion variants, quantified as log_2 FC over the corresponding mock-infected sample. Abbreviations: ISG, interferon-stimulated gene; HVR, hypervariable region; FC, fold change; PHHs, primary human hepatocytes; h p.i., hours post infection.

Supplementary tables:

Table S1: Donor information of primary human hepatocytes used in this study. Abbreviations: N/A, not available; HAV, hepatitis A virus; HBV, hepatitis B virus; HCV, hepatitis C virus; HIV, human immunodeficiency virus; SARS-CoV-2, severe acute respiratory syndrome coronavirus 2.

**Donor Number**	1	2	3
**Donor Lot**	CHM2420-L-HE-Z	HH240404	HH250124
**Sex**	male	female	male
**Age**	54 years	81 years	74 years
**Diagnosis**	Hepatic metastases	Liver metastases with rectal carcinoma	Rectum carcinoma with liver metastasis
**Medical History**	N/A	Hypertension	Hypertension
**Smoker**	N/A	N/A	no
**Therapy**	N/A	Hemihepatectomy right	Hemihepatectomy right
**Medication**	N/A	ASS, Atorvastatin, Ramipril, Amlodipin	Ramipril, Allopurinol
**Serology**	Tested negative for HIV, HBV, SARS-CoV-2 less than 3 months before surgery	HAV, HBV, HCV, HIV 1/2: negative	HAV, HBV, HCV, HIV 1/2: negative

References

1. Shaw AE, Hughes J, Gu Q, Behdenna A, Singer JB, Dennis T, et al. Fundamental properties of the mammalian innate immune system revealed by multispecies comparison of type I interferon responses. PLoS Biol. 2017;15:e2004086. doi:10.1371/journal.pbio.2004086.
